# A complex protein derivative acts as biogenic elicitor of grapevine resistance against powdery mildew under field conditions

**DOI:** 10.3389/fpls.2015.00715

**Published:** 2015-09-18

**Authors:** Andrea Nesler, Michele Perazzolli, Gerardo Puopolo, Oscar Giovannini, Yigal Elad, Ilaria Pertot

**Affiliations:** ^1^Department of Sustainable Agro-Ecosystems and Bioresources, Research and Innovation Centre, Fondazione Edmund MachSan Michele all'Adige, Italy; ^2^Department of Plant Pathology and Weed Research, Agricultural Research Organization, The Volcani CenterBet Dagan, Israel

**Keywords:** biocontrol, *Vitis vinifera*, induced resistance, defense-related genes, gene expression, powdery mildew, protein hydrolysates

## Abstract

Powdery mildew caused by *Erysiphe necator* is one of the most important grapevine diseases in several viticulture areas, and high fungicide input is required to control it. However, numerous synthetic chemical pesticides are under scrutiny due to concerns about their impact on human health and the environment. Biopesticides, such as biogenic elicitors, are a promising alternative to chemical fungicides. Although several studies have reported on effective elicitors against grapevine diseases, their efficacy under field conditions has not been investigated extensively or has occurred at rather limited levels. Our goal was to examine the efficacy of a protein-based composition, namely nutrient broth (NB), against powdery mildew under field conditions and to characterize its mechanism of action. Weekly treatments with NB was highly effective in controlling powdery mildew on grapevine across seasons with different disease pressures. The level of disease control achieved with NB was comparable to standard fungicide treatments both on leaves and bunches across three different years. NB has no direct toxic effect on the germination of *E. necator* conidia, and it activates plant resistance with both systemic and translaminar effect in experiments with artificial inoculation under controlled conditions. NB induced the expression of defense-related genes in grapevine, demonstrating stimulation of plant defense mechanisms, prior to and in the early stages of pathogen infection. NB is a natural derivative from meat and yeast, substances that tend not to raise concerns about toxicological and ecotoxicological properties. NB represents a valid control tool for integrated plant protection programs against powdery mildew, to reduce the use of synthetic pesticides on grapevine.

## Introduction

Synthetic chemical pesticides have contributed significantly to the sizable increase in global production of agricultural goods in recent decades (Hillocks, 2012). On the other hand, the overuse of pesticides has raised concerns about their impact on human health and the environment (Fantke et al., 2012). For these reasons, the European Union (EU), along with many countries around the world, has implemented stringent regulations on the registration of plant protection products, and directed policies toward achieving significant reductions in pesticide use (Hillocks, 2012; Skevas et al., 2013). In addition, several active ingredients are under scrutiny in the EU because of their potential hazards, and a list of candidates for substitution has been drafted (http://ec.europa.eu/food/plant/pesticides/approval_active_substances/). The list contains several molecules (e.g., triazoles), which are currently being used against powdery mildews. However, some natural chemicals used against powdery mildews, such as sulfur, are not without negative effects (e.g., an undesirable reduction of predatory mites, a risk of skin and eye irritation for humans, the potential to cause unpleasant aromas in wine).

Pesticide application on grapevine (*Vitis vinifera*) accounts for a large portion of pesticide use. While viticulture constituted only 3.3% of the total agricultural area of the EU, it accounted for 67% of all fungicides applied to crops in the period from 2001 to 2003 (Qiu et al., 2015). Powdery mildew caused by the fungus *Erysiphe necator* Schwein [synonym *Uncinula necator* (Schw.) Burr.] is one of the most important grapevine diseases worldwide. The pathogen colonizes the epidermal layer of leaves and berries, causing severe yield loss and depreciation of wine quality (Stummer et al., 2003; Calonnec et al., 2004). Most cultivated grapevine varieties are susceptible to powdery mildew and growers need to apply fungicides frequently (every 7–10 days in seasons with high disease pressure), with a dramatic increase in production costs and impacts on human health and the environment (Jones et al., 2014). In California, for example, 20% of total costs associated with wine grape production is directly related to powdery mildew control (Fuller et al., 2014). No commercially relevant resistant cultivars are currently available, and biological alternatives have shown only limited efficacy under field conditions (Crisp et al., 2006a,b; Gadoury et al., 2012). Optimizing dosages and the timing of pesticide treatments as part of integrated pest management programs is a move toward reducing massive and widespread use of fungicides. However, there is also an urgent need to find feasible alternatives to chemical fungicides for controlling grapevine powdery mildew.

To find sustainable approaches to manage the disease, studies have increasingly targeted exogenous molecules and living microorganisms that induce grapevine defense responses (elicitors; Delaunois et al., 2014). The use of elicitors is based on the concept that plants use sophisticated mechanisms to defend against pathogen attacks (Panstruga et al., 2009). Plants can recognize specific pathogen- or microbe-associated molecular patterns (PAMPs or MAMPs) and activate specific pathways through PAMP- or MAMP-triggered immunity (Jones and Dangl, 2006). In addition, damage to plant cells from pathogens can release endogenous damage-associated molecular patterns (DAMPs) that act as warning signals (Wu et al., 2014). DAMPs trigger or amplify plant defense responses and initiate processes that restore plant homeostasis and prepare adjacent tissues for invader perception (Heil and Land, 2014). When specific transmembrane receptors recognize a threat, plant cells react quickly, generating reactive oxygen species, activating protein kinases, and expressing plant defense genes that produce pathogenesis related (PR) proteins and phytoalexins (Macho and Zipfel, 2014; Wu et al., 2014). In addition to reactions directly aimed at impeding local growth of the pathogen, systemic resistance can also be induced in the plant (Pieterse et al., 2009) by complex cross-talk among salicylic acid (SA), jasmonic acid (JA), ethylene (ET), and other plant hormones (Robert-Seilaniantz et al., 2011).

Elicitors can be derived from biological origins or synthetic analogs of plant signaling molecules (Wiesel et al., 2014), and several of them have been tested on grapevine. Bacterial elicitors, such as flagellin and harpin, stimulated grapevine defense responses (Chang and Nick, 2012; Trdá et al., 2014). Likewise, rhamnolipids and ergosterol triggered defense responses against *Botrytis cinerea* (Laquitaine et al., 2006; Varnier et al., 2009) and chitin derivatives induced resistance against *B. cinerea* and *Plasmopara viticola* (Repka, 2001; Aziz et al., 2006; Trotel-Aziz et al., 2006). Native or sulfated oligoglucuronans (Caillot et al., 2012) and elicitin from *Pythium oligandrum* (Mohamed et al., 2007) induced resistance against *B. cinerea. Trichoderma harzianum* T39 induced resistance against downy mildew (Perazzolli et al., 2008, 2011, 2012), as well as the β-1-3-glucan laminarin (Aziz et al., 2003; Trouvelot et al., 2008; Gauthier et al., 2014) and oligogalacturonides (Aziz et al., 2004). Few reports have characterized the efficacy of elicitors for controlling of *E. necator*. Particularly, an optimized chitosan formulation (Iriti et al., 2011) and a complex of chitosan fragments (COS-OGA) reduced powdery mildew severity in grapevine (van Aubel et al., 2014). Likewise, an extract from the green macroalga *Ulva armoricana* controlled powdery mildew (Jaulneau et al., 2011) by inducing plant defense through the JA pathway (Jaulneau et al., 2010). Moreover, knotweed extracts (Milsana) were reported as resistance inducers with moderate efficacy against powdery mildew (Delaunois et al., 2014). Applications of milk, whey and potassium bicarbonate reduced powdery mildew severity (Crisp et al., 2006b), but their efficacy under field conditions was affected by the extent of spray coverage (Crisp et al., 2006a), indicating scarce systemic efficacy.

Plant hormones and synthetic analogs of signaling molecules can also stimulate plant defense and their efficacy against *E. necator* has been explored. Induction of defense responses against grapevine powdery mildew was demonstrated by treatments with ET and SA analogs (Belhadj et al., 2008; Dufour et al., 2013). In particular, the ET-releasing ethephon protected detached grapevine leaves and foliar grapevine cuttings against *E. necator* by inducing defense-related genes (Belhadj et al., 2008). The SA analog benzothiadiazole (BTH) activated grapevine resistance against powdery mildew by inducing *PR-1, PR-3, PR-10*, stilbene synthase (*STS*) and leucoanthocyanidin dioxygenase (*LDOX*) genes both before and after *E. necator* inoculation (Dufour et al., 2013). Moreover, a commercial product based on phosphorous acid with oligoelements (Trafos Mg-Ca-Si) was reported as resistance inducer with moderate efficacy against grapevine powdery mildew (Delaunois et al., 2014). Elicitors can be distinguished according to their chemical nature, and proteins, peptides, protein lysates and protein derivatives represent a wide category of plant resistance inducers (Albert, 2013). Protein hydrolysates from soybean and casein reduced downy mildew symptoms on grapevine by inducing defense-related genes and phytoalexin production (Lachhab et al., 2014). Likewise, a composition based on protein extracts and hydrolysates was patented for the control of powdery mildews on a number of crops, including grapevine (Pertot and Elad, 2009).

To date, a number of studies have addressed the development of elicitors on grapevine, but despite initial enthusiasm justified by reproducible efficacy under lab or greenhouse conditions, they have almost always failed to sufficiently control grapevine disease when applied under field conditions (Delaunois et al., 2014). The purpose of the current study was to demonstrate the efficacy of a complex protein-based composition of meat and yeast extracts against *E. necator* under field conditions. A further aim was to dissect its mechanism of action to further develop a robust elicitor for use in integrated pest management programs of grapevine.

## Materials and methods

### Evaluation of the efficacy of nutrient broth against grapevine powdery mildew under field conditions

Field trials were carried out in 2010, 2011 and 2013 at S. Michele all'Adige (latitude: N46.184391, longitude: E11.124499, altitude: 228 m) in a vineyard (grapevine cultivar Mittervernatsch grafted on Teleki 5C) planted in 1997 and trained into a pergola trentina trellis system (distance of plants: 3.0 m between rows × 0.8 m on the row). No pesticides were used in the experimental vineyard, except for copper hydroxide (Kocide 3000 at 2.5 kg/ha; DuPont de Nemours) sprayed at 7–10 day intervals depending on the weather conditions from the beginning of May to end of July to prevent downy mildew infections.

The nutrient broth (NB) was composed of 0.4 g/l meat extract (Fluka Analytical, Sigma-Aldrich, St. Louis, MO, USA), 0.7 g/l yeast extract (Fluka Analytical) and 1.9 g/l peptone from meat (Fluka Analytical). This composition was selected as the most effective against powdery mildew from a list of laboratory protein extracts commonly used as nutritional factors in microbiological media (peptone, beef extract, yeast extract, tryptone and malt extract; Fluka Analytical). In high throughput preliminary experiments on zucchini (*Cucurbita pepo* cv. Afrodite) plants, the protein extracts were tested against powdery mildew (*Podosphaera xanthii*) at 5.0 g/l; in addition NB was also tested at 0.5 and 2.0 g/l. Briefly, zucchini plants were grown in individual 2.5 l-pots containing a mixture of peat and pumice (3:1). Plants with two fully developed leaves were treated with a hand sprayer, allowed to dry, and inoculated with a water suspension of *P. xanthii* conidia (1 × 10^5^ conidia/ml). Four replicates (plants) were analyzed for each treatment and powdery mildew severity was scored at 14 days post-inoculation (dpi) on all leaves by assessing the percentage of infected leaf area covered by white powdery mildew sporulation according to the standard guidelines of the European and Mediterranean Plant Protection Organization (EPPO, 1990). The efficacy of each treatment was calculated according to the following formula:

$$\begin{array}{l} \text{Efficacy}=\frac{\left(\text{SC}-\text{ST}\right)}{\text{SC}}\times 100 \end{array}$$

Where SC corresponds to disease severity in control plants and ST corresponds to disease severity in plants treated with a tested molecule.

For field trials in the vineyard, the dosage of NB used in the first year (2010) was 5.0 g/l, corresponding to 5.0 Kg/ha. Since slight phytotoxic effects were observed on some leaves at the end of the season (Figure S1), the NB dosage was decreased to 3.0 g/l in the second (2011) and third (2013) years. As untreated control and standard fungicide treatment, plants were sprayed with water (control) or with 5 g/l of wettable sulfur according to the manufacturer's dosage instruction for intermediate infection pressure of *E. necator* (Zolvis 80, Manica, Italy), respectively. Treatments were applied weekly with a motorized backpack mist blower (450, Solo, USA). A randomized complete block design with three replicates of eight plants each per treatment was used. Plants were assessed visually each week using 40 and 20 randomly chosen leaves and bunches per replicate, respectively. Disease severity was assessed as a percentage of infected leaf/bunch area covered by white powdery mildew sporulation, and the disease incidence was calculated as percentage of infected leaves or bunches showing white powdery mildew sporulation, according to the EPPO standard guidelines (EPPO, 1988). To evaluate the cumulative impact on the photosynthetic surface throughout the entire season, the development of the disease in terms of severity and incidence was assessed as the area under the disease progress curve (AUDPC) using this formula:

$$\begin{array}{l} AUDPC=\sum \frac{\left(X_{i}+X_{i+1}\right)}{2}\left(t_{i+1}-t_{i}\right) \end{array}$$

Where *X*_*i*_ corresponds to either disease severity or incidence (%) at assessment *i, X*_*i*__+1_ corresponds to either the severity or incidence (%) at subsequent assessment *i* + 1, and (*t*_*i*__+1_ − *t*_*i*_) corresponds to the number of days between the two consecutive assessments. Temperature and rain during the season were recorded at a nearby meteorological station (http://meteo.iasma.it/meteo/).

### Assessment of the direct toxic effect on the germination of *Erysiphe necator* conidia

To evaluate the direct effect of NB on the germination of *E. necator* conidia, leaf disks (19 mm diameter) were cut from the third and fourth leaves (starting from the apical meristem) of healthy grapevine plants (cv. Pinot noir grafted on Kober 5BB) grown under greenhouse conditions. Leaves were surface sterilized by incubation in 1% hypochlorite for 10 min and rinsed three times by incubation in sterile water for 5 min under orbital shaking at 80 rpm (Bills et al., 2007). Leaf disks were placed (adaxial surface uppermost) onto wet sterilized filter paper (three foils) in Petri dishes. Leaf disks were then homogenously sprayed with water, as untreated control, with 3.0 g/l NB or 5.0 g/l of sulfur as a standard fungicide, using a small hand sprayer. Leaf disks were dried under a laminar hood for 1 h, and conidia were brushed gently on them with a paint brush from young leaves carrying freshly sporulation of *E. necator* at 14 dpi. The *E. necator* inoculum was obtained from infected leaves of untreated vineyards in northern Italy (Trentino region) and was maintained by subsequent inoculations on grapevine plants (cv. Pinot noir grafted on Kober 5BB) under greenhouse conditions at 25 ± 1°C with 80 ± 10% relative humidity (RH). Inoculated leaf discs were incubated for 44 h at 23 ± 1°C in Petri dishes (RH close to 99%) to allow conidia germination. In order to assess germination, conidia were removed from the leaf disc surface using a piece of transparent adhesive tape (2 × 3 cm) and stained with a drop of the cotton blue staining solution according to Peries (1962). The percentage of germinated conidia was assessed by counting under a light microscope and conidia were scored as germinated when their germ tube length was greater than their lateral radius (Pertot et al., 2007). Four replicates of three disks were assessed for each treatment by counting 50 conidia for each leaf disk, and the experiment was carried out twice.

### Assessment of induced resistance and gene expression analysis

Since grapevine leaves rapidly develop ontogenic resistance to powdery mildew under greenhouse conditions, the level of systemic and local resistance activated by NB was tested on zucchini plants with the same methodology used for the preliminary assessment of the efficacy of the protein extracts. In this experiment, zucchini plants with at least eight developed leaves were treated with NB or water (control) on the first four basal leaves to test the local (treated basal leaves) and systemic (untreated apical leaves) effect. Plants were also treated on the abaxial surface of each leaf to analyze the translaminar effect on the adaxial surface. Powdery mildew inoculation of zucchini plants was carried out as described above and disease severity was assessed 14 dpi on the adaxial surface of each leaf, and scores of basal (local effect) and apical (systemic effect) leaves were analyzed separately.

Two-year-old grapevine plants (cv. Pinot noir grafted onto Kober 5BB) were used to assess the level of disease reduction and the expression of defense-related genes. Plants were grown in individual 2.5 l-pots containing a mixture of peat and pumice (3:1) under greenhouse conditions at 25 ± 1°C with a photoperiod of 16 h light and RH of 60 ± 10% for 2 months. Plants were sprayed with water, as untreated control, 3.0 g/l of NB, or 5.0 g/l of sulfur, as standard fungicide. Treatments were repeated daily for three consecutive days at 1, 2, and 3 days before *E. necator* inoculation to maximize the phenotypic response of grapevine induced resistance (Perazzolli et al., 2008). Treatments were applied with a compressed-air hand sprayer to the abaxial and adaxial surfaces of all leaves, and 20–30 ml was applied to each plant, depending on the number of leaves, in order to achieve homogenous distribution. Nine replicates (plants) per treatment were analyzed in a randomized complete block design, and the experiment was carried out twice. One day after the last treatment, all leaves of each plant were inoculated with dry conidia of *E. necator* maintained by subsequent inoculations on grapevine under greenhouse conditions as described above. Conidia were brushed gently with a paint brush from infected young leaves (14 dpi) carrying freshly sporulation of *E. necator* onto the target leaves (Blaich et al., 1989). Inoculated plants were incubated overnight in the dark at 25 ± 1°C with 95 ± 4% RH and then kept under greenhouse conditions at 25 ± 1°C with 80 ± 10% RH for 13 days. Disease severity was assessed visually on each leaf according to the EPPO standard guidelines (EPPO, 1988).

For gene expression analysis, leaf samples were collected immediately before (0), at one (1), six (6) and 13 dpi with powdery mildew. For each treatment, leaf samples were collected from three different plants (replicates) at each time point. Each sample comprised three half-leaves from the same plant, and only leaves of the fourth, fifth, and sixth node starting from the apical meristem were collected, pooled, immediately frozen in liquid nitrogen and stored at −80°C. Total RNA extraction, cDNA synthesis and quantitative real-time PCR (RT-qPCR) reactions were carried out as described Banani et al. (2014) using specific primers (Table S1). Briefly, each sample was examined in three technical replicates, and dissociation curves were analyzed to verify the specificity of each reaction. Cycle threshold (Ct) values were extracted with the Light Cycler 480 SV1.5.0 software (Roche) using the second derivative calculation and LinReg 11.1 software was used to calculate reaction efficiency (Ruijter et al., 2009). The relative expression of each gene was obtained according to the Pfaffl equation (Pfaffl, 2001), using leaves of control plants at 0 dpi as the calibrator. The γ-chain elongation factor 1 gene (EF1-γ) was used as reference gene with constitutive expression for data normalization (Dufour et al., 2013). For each sample, mean gene expression and standard error values were calculated based on three replicates, and two independent repetitions of the experiment were analyzed.

### Statistical analysis

Data were analyzed using Statistica 9 software (StatSoft, Tulsa, OK, USA). Disease severity and incidence scores on grapevines were normalized by transformation (with arcsin for the field experiments and log_10_ for the greenhouse experiments). Fold change values of gene expression analysis were normalized by the equation Y = log_10_ (1 + x) (Casagrande et al., 2011). AUDPC data and efficacy scores were normalized by square root and arcsin transformation, respectively. After validation of normal distribution (K-S test, α < 0.05) and variance homogeneity (Levene's test, α < 0.05) of the data, analysis of variance (ANOVA) was carried out and the Tukey's HSD test (α = 0.05) was applied to detect significant differences among treatments. An F-test was used to demonstrate non-significant treatment-experiment interactions (α > 0.05). A *t*-test (α = 0.05) was used to analyze severity scores of NB-treated and control plants in the experiments of translaminar and systemic effect.

## Results

### Evaluation of the efficacy of nutrient broth against grapevine powdery mildew under field conditions

**Figure 1:**
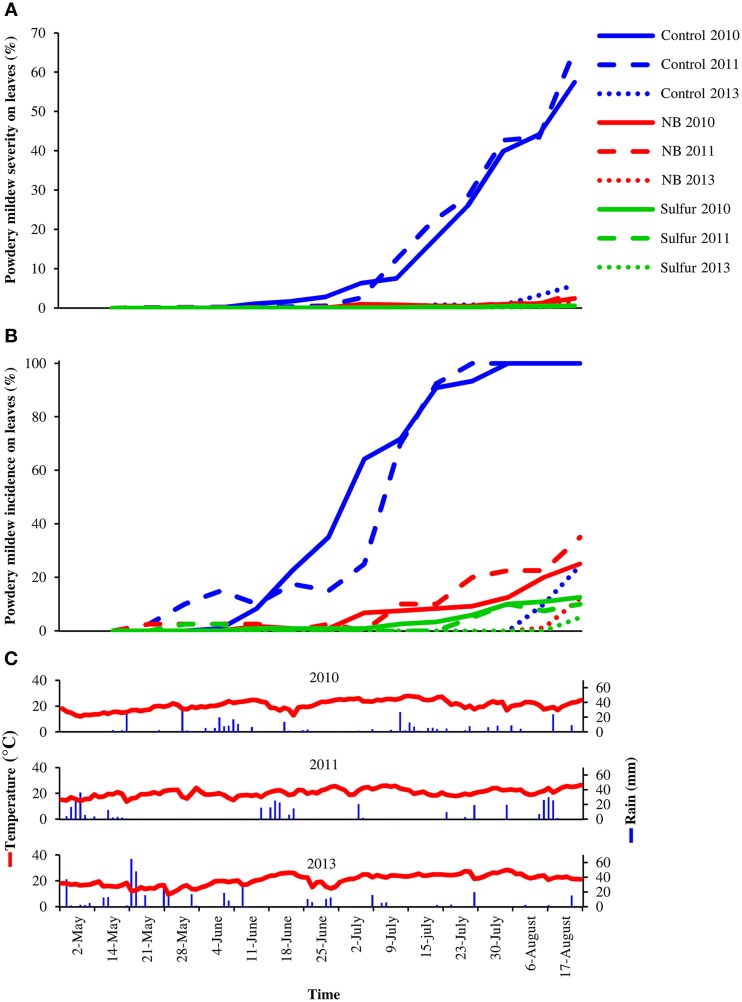
**Effect of nutrient broth against powdery mildew on grapevine leaves under field conditions**. Powdery mildew severity **(A)** and incidence **(B)** was assessed on leaves of grapevine plants treated with water (Control), nutrient broth (NB) or sulfur (Sulfur), as standard fungicide, under field conditions in three different seasons (2010, 2011, and 2013). Treatments were applied weekly from the beginning of May to the middle of August. Disease severity and incidence were assessed weekly as percentage of adaxial leaf area covered by white sporulation and percentage of leaves showing symptoms by scoring 40 leaves per replicate, respectively. The mean severity and incidence scores of three replicates (eight plants each) are presented for each treatment. The temperature and rain during the seasons were recorded by a meteorological station **(C)**. The dosage of NB was 5.0 g/l in 2010, and was reduced to 3.0 g/l in 2011 and 2013 to avoid phytotoxicity.

**Figure 2:**
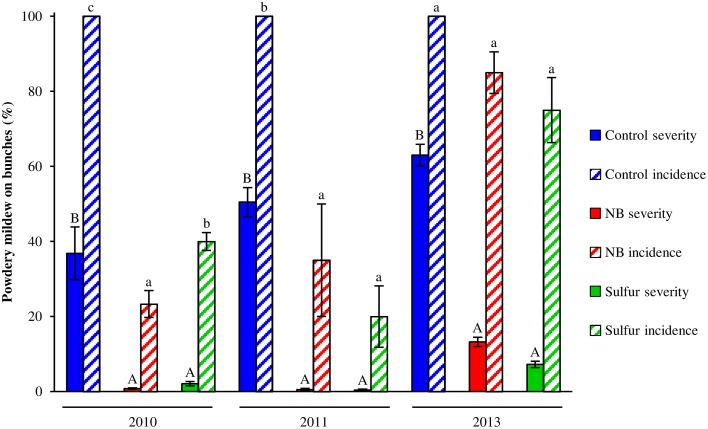
**Effect of nutrient broth against powdery mildew on grape bunches under field conditions**. Powdery mildew severity and incidence was assessed on bunches of grapevines treated with water (Control), nutrient broth (NB) or sulfur (Sulfur), as standard fungicide, under field conditions in three different seasons (2010, 2011, and 2013). Treatments were applied weekly from the beginning of May to the middle of August. Disease severity and incidence were assessed weekly as percentage of bunch area covered by white sporulation and percentage of bunches showing symptoms by scoring 20 bunches per replicate, respectively. The mean severity and incidence scores and the standard errors of three replicates (eight plants each) are presented for each treatment. For each year, uppercase and lowercase letters indicate significant differences among treatments according to Tukey's HSD test (α = 0.05) of disease severity and incidence, respectively.

Laboratory protein extracts were tested against powdery mildew on zucchini plants. Although the efficacy was statistically comparable to 2.0 g/l NB and 5.0 g/l beef extract, 5.0 g/l NB provided the greatest reduction of powdery mildew symptoms (Figure S2) and this formulation was used in the field trials. In 2010 and 2011, primary infections occurred very early in the season and the first signs of the disease were observed on June 6 and May 19, respectively. The disease intensified very quickly in these two seasons, resulting in high levels of severity and incidence on leaves of control plants (Figure 1). In 2013, due to unsuitable conditions for the disease (high mean daily temperatures and low precipitation starting in June), powdery mildew developed slowly and very late in the season and symptoms were observed mainly on bunches rather than leaves starting on July 7 on control plants. The onset of the disease occurred almost at the same time on leaves of control, NB- and sulfur-treated plants in the 3 years tested. However, disease progress during the season was slower in NB- and sulfur-treated plants compared to control plants, resulting in significantly lower levels of powdery mildew severity and incidence during the season. AUDPC calculated for severity and incidence on leaves of NB-treated plants was comparable to sulfur-treated plants and significantly lower than that of control plants across the 3 years (Table 1). Likewise, powdery mildew severity on grape bunches was significantly lower on NB-treated plants compared to control plants at the end of each of the 3 years tested, and was comparable to that of sulfur-treated plants (Figure 2). Disease incidence was significantly reduced by NB treatments on bunches in 2010 and 2011, but not in 2013. Except for the slight phytotoxic effects of 5 g/l NB at the end of the 2010 season, no negative effect of 3 g/l NB on leaf morphology, shoot growth and fruit yield were visible at the end of the 2011 and 2013 seasons (Figure S1).

**Table 1:** **Area under the disease progress curve (AUDPC) of grapevine powdery mildew under field conditions**.

**Year**	**Treatment**	**AUDPC on severity**	**AUDPC on incidence**
2010	Control	1667.6±312.6b	5544.2±233.6b
	NB	16.2±0.9a	398.3±58.1a
	Sulfur	54.9±15.7a	726.6±235.3a
2011	Control	1506.9±101.5b	4842.5±98.8b
	NB	11.5±6.3a	266.5±136.1a
	Sulfur	46.0±13.0a	926.5±301.3a
2013	Control	101.1±40.1b	756.8±23.3b
	NB	7.3±2.5a	120.0±13.8a
	Sulfur	3.5±3.5a	29.9±7.7a

*Area under the disease progress curve (AUDPC) was calculated for powdery mildew severity and incidence on leaves of grapevines treated with water (Control), nutrient broth (NB) or sulfur (Sulfur), as standard fungicide, under field conditions in three different seasons (2010, 2011, and 2013). Treatments were applied weekly from the beginning of May to the middle of August. Disease severity and incidence were assessed weekly as percentage of adaxial leaf area covered by white sporulation and percentage of leaves showing symptoms by scoring 40 leaves per replicate, respectively. The mean AUDPC and standard error values of three replicates (eight plants each) are presented for each treatment. For each year, different letters indicate significant differences of disease incidence and severity among treatments according to Tukey's HSD test (α = 0.05)*.

### Characterization of the mechanism of action of nutrient broth: direct effect on the germination of conidia of *Erysiphe necator*

No direct effect of NB on *E. necator* conidia germination was demonstrated, and the percentage of germinated conidia on control (50 ± 2%) and NB-treated (44 ± 1%) leaf disks was comparable in both the experiments (*F*-test, *p* = 0.46). On the other hand, the treatment with sulfur reduced the percentage of conidia germination by 70 ± 2% compared to control leaf disks (Figure S3).

### Characterization of the mechanism of action of nutrient broth: assessment of induced resistance and gene expression analysis

**Figure 3:**
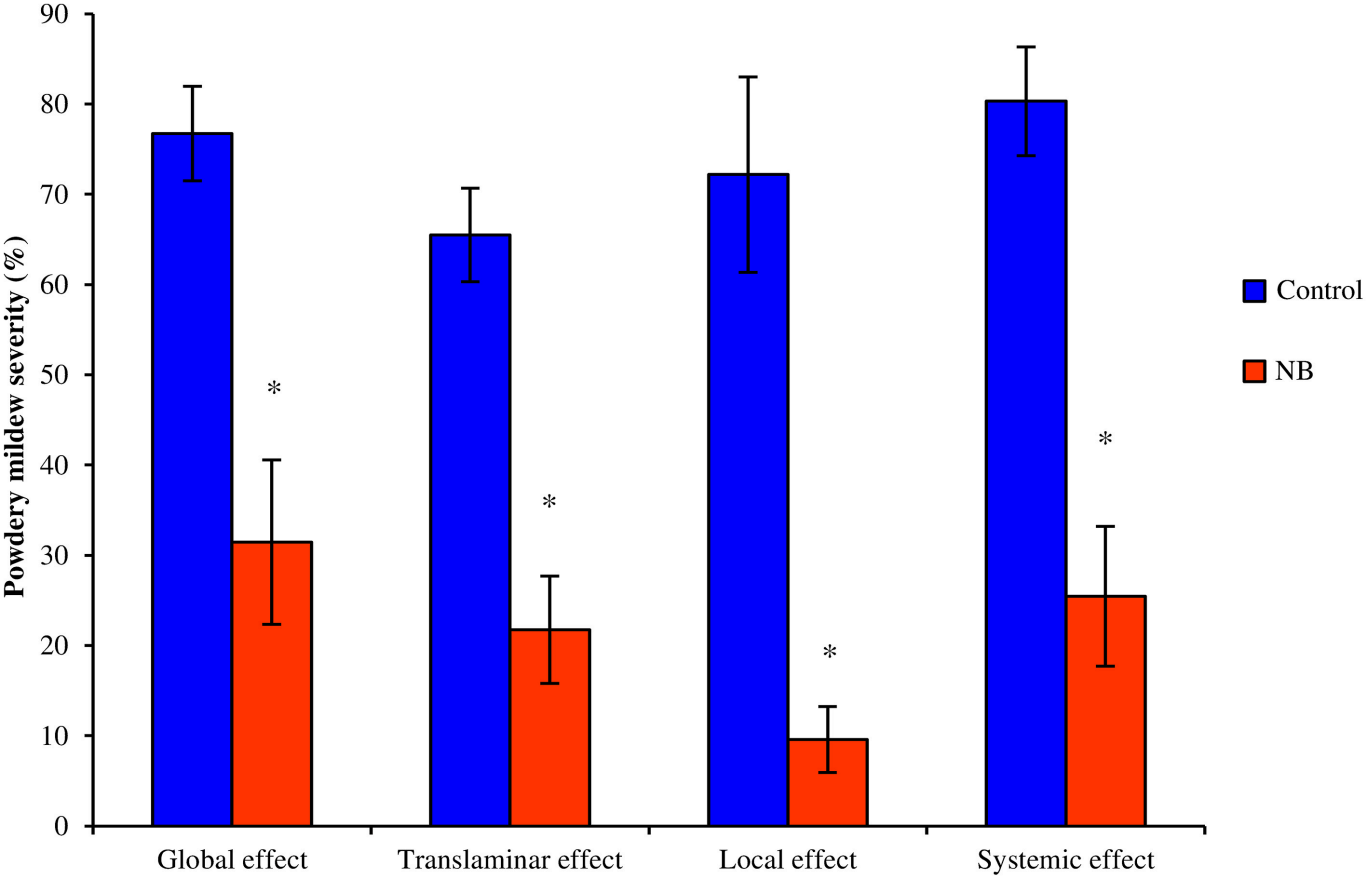
**Effect of nutrient broth against powdery mildew on zucchini plants under greenhouse conditions**. Powdery mildew severity were evaluated on zucchini plants treated with nutrient broth (NB) or water (Control). Treatments were sprayed to both surfaces of each leaf (Full treatment), to the abaxial surface of each leaf (Abaxial treatment) or to the first four basal leaves (Basal treatment). Disease severity was assessed as the percentage of adaxial leaf area covered by white sporulation 14 days post-inoculation. Global and translaminar effect was calculated on all leaves of full and abaxial treatment, respectively. For basal treatment, scores of treated basal (Local effect) and untreated apical (Systemic effect) leaves were analyzed separately. *F*-test revealed non-significant differences between experiments (*P* > 0.05) and data from two experimental repetitions were pooled. The mean severity and standard error values of eight replicates (potted plants) of two experiments are presented for each treatment. For each treatment, asterisks indicate significant differences between NB-treated and control plants according to *t*-test (α = 0.05)

Foliar applications of NB on leaves of zucchini plants significantly reduced powdery mildew symptoms (Figure 3). NB applied to the first four basal leaves reduced disease symptoms on untreated apical leaves, demonstrating the activation of systemic resistance. Likewise, NB treatment of the abaxial leaf surface significantly reduced powdery mildew symptoms on the adaxial surface, demonstrating a translaminar effect.

**Figure 4:**
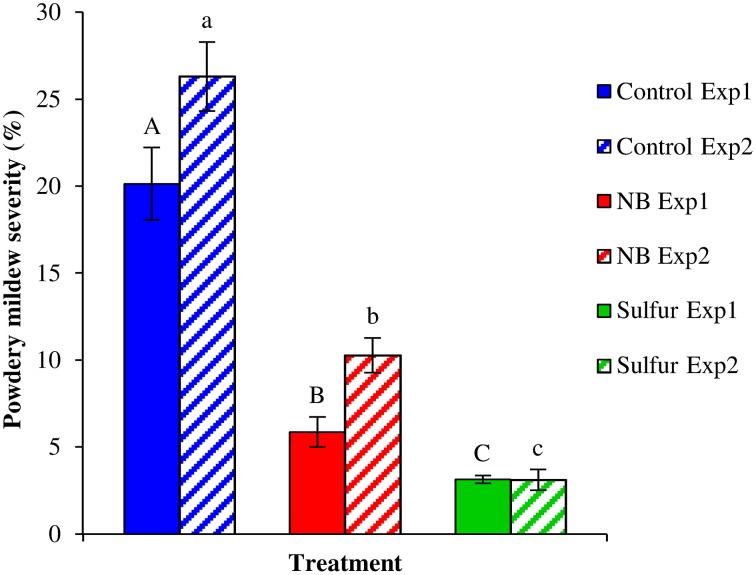
**Effect of nutrient broth against powdery mildew on grapevine plants under greenhouse conditions**. Powdery mildew severity was assessed on grapevine leaves treated with water (Control), nutrient broth (NB) or sulfur (Sulfur), as standard fungicide for 3 days before pathogen inoculation. Disease severity was assessed as percentage of adaxial leaf area covered by white sporulation 13 days post-inoculation, and two independent experiments were carried out under greenhouse conditions. The mean severity and standard error values of nine replicates (potted plants) are presented for each treatment and experiment. Uppercase and lowercase letters indicate significant differences among treatments according to Tukey's HSD test (α = 0.05) in Experiments 1 (Exp1) and 2 (Exp2), respectively.

**Figure 5:**
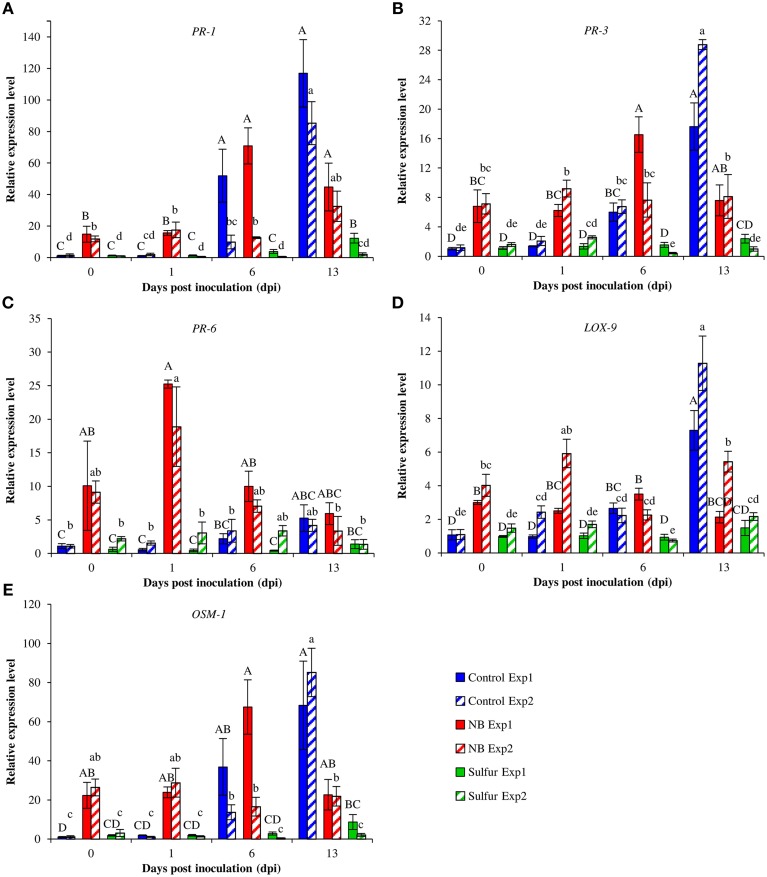
**Effect of nutrient broth on gene expression in grapevine plants under greenhouse conditions**. Relative expression levels of genes encoding the pathogenesis-related protein 1 (*PR-1*; **A**), *PR-3* **(B)**, *PR-6* **(C)**, lipoxygenease 9 (*LOX-9;* **D**) and osmotin 1 (*OSM-1*; **E**). Grapevine plants were treated with water (Control), nutrient broth (NB) or sulfur (Sulfur), as standard fungicide, for 3 days before pathogen inoculation. Leaf samples were collected just before inoculation (0), at one (1), six (6) and 13 days post-inoculation (dpi). Relative expression levels were calculated using the γ-chain elongation factor 1 gene (*EF1*-γ) as constitutive gene for normalization, and data were calibrated on control plants collected at 0 dpi. For each time point, mean levels of relative expression and standards errors from three replicates (potted plants) are presented for each treatment and experiment. For each gene, uppercase and lowercase letters indicate significant differences among treatments and time points according to Tukey's HSD test (α = 0.05) in Experiments 1 (Exp1) and 2 (Exp2), respectively.

Foliar applications of NB significantly reduced powdery mildew symptoms of the susceptible cultivar Pinot noir under greenhouse conditions (Figure 4). The two experiments were analyzed separately because a slight effect of the “experiment” factor was present (*F*-test, *p* = 0.03). NB treatment significantly reduced the level of disease severity, with an efficacy greater than 60% in both experiments, and the efficacy of sulfur was greater than 84%. In order to investigate the molecular mechanisms of the grapevine induced resistance, the relative expression levels of five defense-related genes (Table S1) were analyzed by RT-qPCR. The expression of the PR protein 1 (*PR-1*) gene was induced at 6 (more than 9-fold) and 13 (more than 85-fold) dpi of powdery mildew inoculation in control plants (Figure 5A). *PR-1* was induced more than 10-fold in NB-treated plants at 0 and 1 dpi in both the experiments. The expression level of *PR-1* further increased at 6 dpi (70-fold) and 13 dpi (44-fold) in NB-treated plants in experiment 1. In experiment 2, the expression level of *PR-1* at 6 dpi (12-fold) and 1 dpi were comparable, and reached 32-fold at 13 dpi. In both the experiments, the expression level of *PR-1* was greater in NB-treated than in control plants at 0 and 1 dpi, but not at 6 and 13 dpi. The expression level of *PR-1* was not affected by powdery mildew inoculation in sulfur-treated plants at any of the time points tested, and it was slightly induced at 13 dpi in Experiment 1.

The expression of the *PR-3* gene was up-regulated (6-fold) in control plants at 6 dpi, and it was further induced to 17-fold and 28-fold at 13 dpi in experiments 1 and 2, respectively (Figure 5B). NB treatment increased the expression (more than 6-fold) of *PR-3* at 0 and 1 dpi in both the experiments. In experiment 1, the expression of *PR-3* in NB-treated plants reached the highest expression level at 6 dpi (16-fold) and it decreased, although not significantly, at 13 dpi. In sulfur-treated plants, the *PR-3* gene was not significantly modulated across the duration of the experiment.

In control plants, the expression level of *PR-6* was maintained at the basal level at 1 and 6 dpi, and it was slightly induced at 13 dpi (Figure 5C). The *PR-6* expression level was greater in NB-treated than control plants at 1 dpi in both the experiments, and at 0 dpi in experiment 1. The highest level of *PR-6* expression (more than 18-fold) was observed at 1 dpi in NB-treated plants.

The expression of the lypoxygenase 9 (*LOX-9*) gene was slightly increased at 6 dpi and it reached the highest expression level at 13 dpi (more than 7-fold) in control plants (Figure 5D). The NB-treated plants showed induction of *LOX-9* at 0 and 1 dpi. At 6 dpi, the expression levels of *LOX-9* in NB-treated and control plants were comparable, and they were statistically greater than sulfur-treated plants. However, *LOX-9* expression level was greater in control than NB-treated plants at 13 dpi.

The expression level of the osmotin 1 (*OSM-1*) gene remained at the basal level at 0 and 1 dpi in control plants, and it was induced more than 36- and 13-fold at 6 dpi in experiments 1 and 2, respectively (Figure 5E). NB increased more than 22-fold the level of *OSM-1* expression at 0 and 1 dpi in comparison to control plants in both experiments. The expression level of *OSM-1* was comparable in NB-treated and control plants at 6 dpi, and it was 3-fold lower in NB-treated than in control plants at 13 dpi. *OSM-1* expression was not significantly affected by powdery mildew inoculation in sulfur-treated plants, except for a slight increase at 13 dpi in Experiment 1.

## Discussion

Concerns about the impact of synthetic chemical pesticides on human health and environment have grown enormously in the last two decades, especially for crops that require elevated number of treatments, such as grapevine. Several alternatives to pesticide use on grapevine have been proposed, including microbial biological control agents (Elmer and Reglinski, 2006), resistant *Vitis* spp. hybrids (Akkurt et al., 2007), and genetically modified plants (Delaunois et al., 2009). However, none of these solutions has become standard practice for powdery mildew control on grapevine. A promising alternative method is the induction of the grapevine defense responses through elicitors (Delaunois et al., 2014). Several authors have reported the induction of resistance by protein-based elicitors in plants (Albert, 2013; Colla et al., 2014; Lachhab et al., 2014), however the contribution of resistance inducers in plant protection under field conditions has rarely been confirmed or has occurred at rather limited levels (Delaunois et al., 2014). In grapevine, most of the studies have been focused on downy mildew with the aim of finding alternatives to copper treatments (Aziz et al., 2003, 2006; Trouvelot et al., 2008; Dagostin et al., 2011; Perazzolli et al., 2011; Delaunois et al., 2014). Some elicitors and protein derivatives have been tested against grapevine powdery mildew to date (Belhadj et al., 2006, 2008; Crisp et al., 2006a,b; Dufour et al., 2013; van Aubel et al., 2014), but they have not been shown to be capable of directly replacing conventional fungicides in vineyards (Delaunois et al., 2014). This study presents the first proof of consistent efficacy of a protein-based elicitor against powdery mildew of grapevines under field conditions across three seasons with different disease pressures. The level of disease control was very high both on leaves and bunches, an efficacy which is usually rare for a resistance inducer applied alone under field conditions.

Systemic and translaminar effect of NB on zucchini plants indicated that the mechanism of action against powdery mildew is mainly based on the induction of plant resistance rather than a direct toxic effect on conidia. NB did not directly affect the germination of *E. necator* conidia and induced the expression of defense-related genes in grapevine, as reported for soybean and casein hydrolysates against downy mildew (Lachhab et al., 2014). *E. necator* infection is most probably blocked or limited by the defense reaction of the plant, as reported in the post-infection processes of resistant grapevine species (Fung et al., 2008), and further deep histological analyses could elucidate the key steps of fungal growth inhibition in NB-treated plants. In comparison to control plants, expression of *PR-1, PR-3, LOX-9,* and *OSM-1* was induced in NB-treated plants before and at 1 dpi with *E. necator*. At 6 dpi, the expression of *PR-1, PR-6, LOX-9*, and *OSM-1* was comparable in NB-treated and control plants. The expression profiles of *PR* genes suggests that the NB-induced defense is directly activated before inoculation, and it plays a major role in the early stages of powdery mildew infection in limiting colonization of the host. Particularly, *E. necator* conidiospores produced appressoria and secondary hyphae on *V. vinifera* leaves at 1 dpi (Fung et al., 2008) and defense genes were already activated in NB-treated plants at this time point, suggesting an appropriate inhibition of the early phases of fungal penetration. The relevance of a rapid up-regulation of defense-related genes during pathogen infection has also been demonstrated for the response of resistant genotypes against downy mildew (Polesani et al., 2010; Casagrande et al., 2011). Direct up-regulation of defense-related genes before *E. necator* inoculation was also associated with grapevine resistance elicited by ethephon (Belhadj et al., 2008) and BTH (Dufour et al., 2013). In BTH-treated plants, *PR-1, PR-3*, and *LOX-9* were induced before inoculation, while *PR-6* was mainly induced at 1 dpi, depending on the *E. necator* strain (Dufour et al., 2013). In control plants, the defense-related genes tested were induced at 6 and 13 dpi, but not at 1 dpi, suggesting that defense response occurred too late to effectively counteract the pathogen. Biotrophic pathogens circumvent plant defense responses during compatible interaction by the release of effectors into the host tissue (Panstruga, 2003; Schulze-Lefert and Panstruga, 2003), and some of them act as suppressors of basal plant defense (Schulze-Lefert and Panstruga, 2003; Chisholm et al., 2006). No induction of defense related genes was detected in the early stages of *E. necator* infection in the susceptible cultivar, indicating the relevance of the defense pre-activation stimulated by NB treatment. Marker genes of SA and JA pathways, such as *PR-1* and *LOX-9* respectively (Hamiduzzaman et al., 2005), were induced by NB treatment, suggesting activation of complex hormone-mediated signaling pathways. Indeed, both SA and JA signals were recently linked to the defense reaction of resistant grapevine genotypes against powdery mildew (Gao et al., 2014; Weng et al., 2014), and the assessment of plant hormone accumulation after NB treatments could be the subject of further studies to better understand mechanisms of the NB-induced response. Activation of multiple defense pathways suggests broad spectrum activity of NB against different pathogens, particularly those with a biotrophic lifestyle. Two different genera that cause powdery mildew disease were controlled by NB in two different crops, highlighting the strong potential of this natural product against crop diseases.

The protein derivative used in this study is produced for laboratory use using a precisely defined and reproducible process, and its efficacy against powdery mildew is consistent among different batches. NB production processes can be easily replicated at the industrial level using relatively cheap by-products from food industries, and it may represent an additional tool for integrated pest management programs that aim to reduce the use of synthetic chemical pesticides. The advantage of using a protein derivative to induce resistance on plants is the absence of toxicity for mammals and the environment, and the activation of systemic mechanisms that protect plants even if rain washes the product from leaves. Moreover, the absence of negative effects from weekly applications of 3 g/l NB on plant growth and yield indicates minimal risks for grape production and quality. However, attention should be paid to combining this treatment with copper-based products because copper can be partially absorbed by leaf cells in presence of peptides (Pertot et al., 2006), increasing the risk of phytotoxicity.

In conclusion, our results support the use of NB against powdery mildew in grapevine. We have demonstrated the efficacy of NB under field conditions across three different seasons. However, factors such as vegetative cycle, agro-climatic conditions (Delaunois et al., 2014), plant genotypes (Banani et al., 2014) and abiotic stresses (Roatti et al., 2013) could affect the induction of plant resistance and reduce the efficacy of resistance inducers under field conditions. Further studies on the relevance of the vegetative cycle and the physiological status of the plant are required to optimize the efficacy of resistance inducers under field conditions (Delaunois et al., 2014). Protein hydrolysates contain a large variety of active peptides, which could also act as antioxidants and biostimulants (Colla et al., 2014). Particularly, NB contains peptides which could elicit grapevine resistance, as reported for the soybean and casein hydrolysates (Lachhab et al., 2014). NB also contains protein fragments and active enzymes that could be directly recognized as MAMPs (Jones and Dangl, 2006) or be responsible for the release of DAMP signals (Heil and Land, 2014; Wu et al., 2014) that activate plant defenses. Thus, NB composition should be further investigated to identify with more precision the elicitors of plant resistance and to better understand which protein fragments and peptides are active in plant resistance activation.

## Author contributions

AN and MP carried out the real time experiments, analyzed data and wrote the manuscript (AN and MP contributed equally to this work), GP carried out the greenhouse experiments, analyzed the data and edited the manuscript, OG carried out the field experiments, analyzed the data and edited the manuscript, YE contributed to the conception of the work, designed the experiments analyzed the data and edited the manuscript, IP contributed to the conception of the work, designed the experiments, carried out the greenhouse experiments, analyzed the data and wrote the manuscript. All authors have read the manuscript and agree with its content.

### Conflict of interest statement

The authors declare that the research was conducted in the absence of any commercial or financial relationships that could be construed as a potential conflict of interest.
