# Supplementary material for: A complex protein derivative acts as biogenic elicitor of grapevine resistance against powdery mildew under field conditions
Source: Front Plant Sci. 2015 Sep 18;6:715. doi: 10.3389/fpls.2015.00715 (PMC4585195; doi:10.3389/fpls.2015.00715)
Supplement: Supplementary file 1 [file Table1.PDF]

Table S1 | Primer sequences for quantitative real time RT-PCR (RT-qPCR) expression analysis of grapevine genes.

Gene name	Abbreviation	Accession number[a]	Grapevine gene[b]	RT-qPCR primers[c]	
Pathogenesis-related protein 1	*PR-1*	AJ536326	VIT_203s0088g00700	PR-1_For<br>PR-1_Rev	ACTTGTGGGTGGGGGAGAA<br>TGTTGCATTGAACCCTAGCG
Pathogenesis-related protein 3	*PR-3*	VVU97521	VIT_205s0094g00340	PR-3_For<br>PR-3_Rev	TATCCATGTGTCTCCGGTCA<br>TGAATCCAATGCTGTTTCCA
Pathogenesis-related protein 6	*PR-6*	AY156047	VIT_205s0020g05000	PR-6_For<br>PR-6_Rev	ACGAAAACGGCATCGTAATC<br>TCTTACTGGGGCACCATTTC
Lypoxygenase 9	*LOX-9*	AY159556	VIT_214s0128g00780	LOX-9_For<br>LOX-9_Rev	CCCTTCTTGGCATCTCCCTTA<br>TGTTGTGTCCAGGGTCCATTC
Osmotin 1	*OSM-1*	XM_002282928	VIT_202s0025g04310	OSM-1_For<br>OSM-1_Rev	CGCTGCGCTAAAGACTACC<br>AAAAACCTTGAGTAATCTGTAGCA
γ chain elongation factor 1	*EF1-γ*	AF176496	VIT_212s0035g01130	EF1-γ _For<br>EF1-γ _Rev	GAAGGTTGACCTCTCGGATG<br>AGAGCCTCTCCCTCAAAAGG

[a] Accession number of the National Center for Biotechnology Information (NCBI) Gene Bank (www.ncbi.nlm.nih.gov).
[b] Gene identifier of the *V. vinifera* database V2 version (http://genomes.cribi.unipd.it/grape/).
[c] Forward (For) and reverse (Rev) sequences of primer pairs. References of primer pairs are: *PR-1* (Perazzolli et al., 2011); *OSM-1* (Roatti et al., 2013); *EF1-γ, PR-3, PR-6* (Dufour et al., 2013); *LOX-9* (Trouvelot et al., 2008).
