# Supplementary material for: A complex protein derivative acts as biogenic elicitor of grapevine resistance against powdery mildew under field conditions
Source: Front Plant Sci. 2015 Sep 18;6:715. doi: 10.3389/fpls.2015.00715 (PMC4585195; doi:10.3389/fpls.2015.00715)
Supplement: Supplementary file 2 [file Image1.PDF]

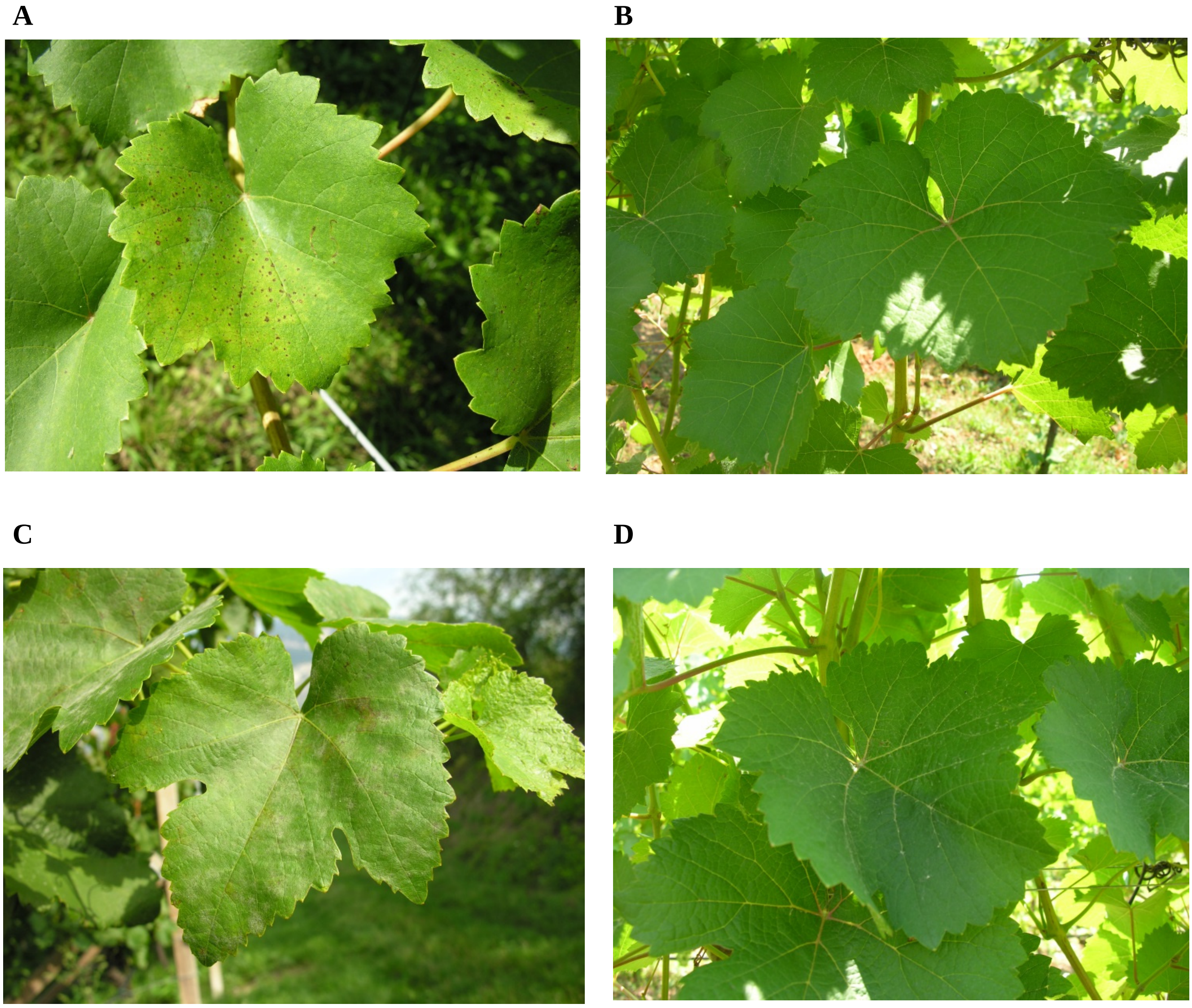

FIGURE S1 | Examples of grapevine plants treated under field conditions. Slight phytotoxicity was observed on leaves of plants treated with nutrient broth at 5 g/l under field conditions at the end of the season in 2010 (**A**). No visible phytotoxic effects of 3 g/l NB were observed in 2011 or 2013 (**B**), powdery mildew infections were visible on leaves of H_2O-treated plants (**C**), and physiology of leaves of NB-treated plants (**B**) was comparable to H_2O- (**C**) and sulfur- (**D**) treated plants.
