# Supplementary material for: A complex protein derivative acts as biogenic elicitor of grapevine resistance against powdery mildew under field conditions
Source: Front Plant Sci. 2015 Sep 18;6:715. doi: 10.3389/fpls.2015.00715 (PMC4585195; doi:10.3389/fpls.2015.00715)
Supplement: Supplementary file 3 [file Image2.PDF]

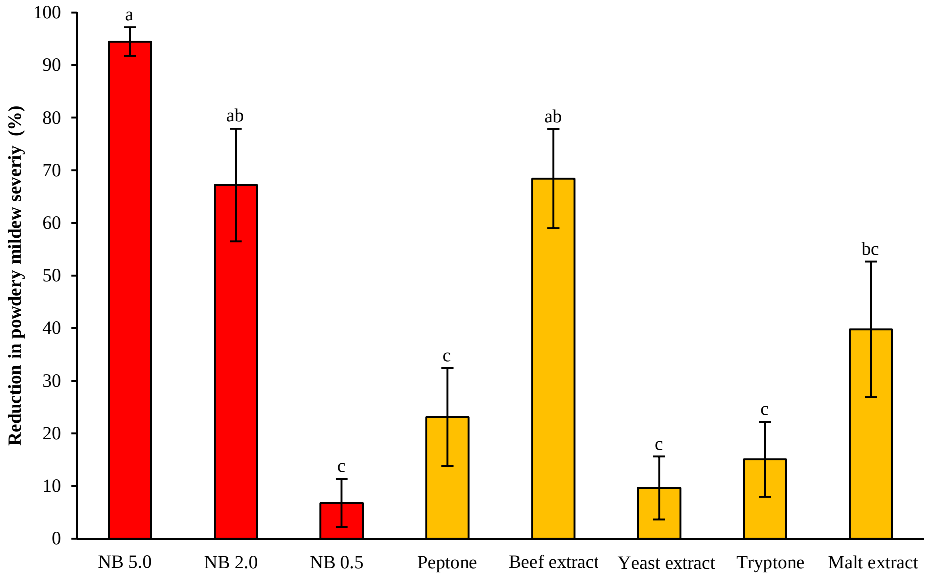

FIGURE S2 | Efficacy of different protein extracts and derivatives against powdery mildew on zucchini plants. Plants were treated with the different compounds one day before inoculation with a water suspension of *Podosphaera xanthii* conidia. Three concentrations (5.0, 2.0 and 0.5 g/l) of nutrient broth (NB) and 5.0 g/l of different protein extracts commonly used as nutritional factors in microbiological media (peptone, beef extract, yeast extract, tryptone and malt extract) were tested. Disease severity was assessed as percentage of adaxial leaf area covered by white sporulation 14 days after inoculation. Reduction (percentage) of disease severity was calculated with respect to water-treated plants used as control, according to the following formula: (disease severity in control plants – disease severity in plants treated with a tested molecule) / (disease severity in control plants) × 100. F-test revealed non-significant differences between experiments ($P > 0.05$) and data from two experimental repetitions were pooled. For each treatment, mean efficacy scores and standard errors of eight replicates (potted plants) of two experiments are presented. Different letters indicate significant differences among treatments according to Tukey's HSD test ($\alpha = 0.05$).
