# Supplementary material for: A complex protein derivative acts as biogenic elicitor of grapevine resistance against powdery mildew under field conditions
Source: Front Plant Sci. 2015 Sep 18;6:715. doi: 10.3389/fpls.2015.00715 (PMC4585195; doi:10.3389/fpls.2015.00715)
Supplement: Supplementary file 4 [file Image3.PDF]

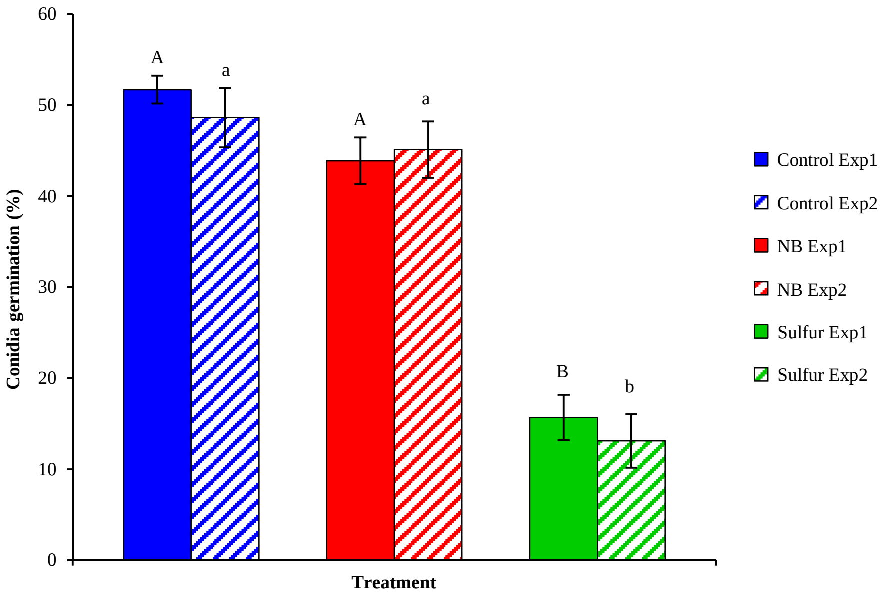

Figure S3 | Direct effect of nutrient broth on *Erysiphe necator* conidia germination. Conidia germination of *E. necator* was assessed on grapevine leaf disks treated with 3.0 g/l nutrient broth (NB), water (Control) or sulfur (Sulfur), as standard fungicide, in two independent experiments (Exp1 and Exp2). The mean percentages of germinated conidia and the standard errors of four replicates (three leaf disks each) are presented for each treatment. An *F*-test revealed non-significant treatment-experiment interactions (p = 0.46). Uppercase and lowercase letters indicate significant differences among treatments according to Tukey's HSD test (α = 0.05) in experiment 1 (Exp1) and 2 (Exp2), respectively.
